# Religious beliefs, social pressure, and stigma: Rural women’s perceptions and beliefs about vasectomy in Pwani, Tanzania

**DOI:** 10.1371/journal.pone.0230045

**Published:** 2020-03-20

**Authors:** Eunice S. Pallangyo, Agnes Cyril Msoka, Sharon Brownie, Eleanor Holroyd

**Affiliations:** 1 Aga Khan University, School of Nursing and Midwifery, Dar es Salaam, Tanzania; 2 Wintec, Centre for Health & Social Practice, Hamilton, New Zealand; 3 School of Medicine Griffith University, Gold Coast, Australia; 4 Green Templeton College, Oxford University, Oxford, England, United Kingdom; 5 Aga Khan University School of Nursing and Midwifery, Kampala, Uganda; University of California Los Angeles, UNITED STATES

## Abstract

Despite being a reliable and cost effective family planning method, vasectomy remains underutilized in many low resource settings such as East Africa. We explored rural women’s perceptions and beliefs regarding barriers to vasectomy use in the low resource setting of Pwani, Tanzania. The qualitative study used in-depth semi-structured interviews to obtain data. Purposive sampling was used to recruit 20 married/cohabiting women with two or more children. Thematic analysis guided the data analysis, with qualitative data reporting informed by COREQ guidelines. Most participants were Muslim and had between two and six children. Most had completed primary-level education and were engaged in small-scale farming. We extracted three main themes with associated sub-themes:1) *lack of education*, which included men’s education levels and inadequate knowledge and misinformation 2) *religious beliefs*, *social pressure and stigma*, which included community stigma and the belief that vasectomy was not good for men with multiple wives; and 3) *promoting men’s involvement in family planning* which included educating men and the women’s perceived role in promoting vasectomy. Participating women perceived vasectomy uptake to be affected by a lack of low knowledge (among men, women, and the community), misinformation, and various sociocultural barriers. Efforts to promote vasectomy and male involvement in reproductive health services should be directed to addressing deeply-rooted sociocultural barriers. Women may have an essential role in encouraging their partners’ vasectomy uptake. In addition, engaging couples in family planning education is critical to enhance knowledge. Ideally, such community based education should be conducted in partnership with communities and healthcare providers.

## Background

Family planning is highlighted by the World Health Organization Sustainable Development Goals (Target 3.7) as a crucial component to achieve universal access and rights to sexual and reproductive healthcare services [1]. Addressing barriers to family planning use also requires the education and involvement of men to better support shared decision making in family matters, particularly where gender inequalities persist and decision-making power is predominantly held by men [2].

Increased use of modern family planning methods is reported in high-income countries, but uptake remains low in low- and middle-income countries [3]. Correspondingly, maternal and neonatal deaths in low- and middle-income countries constitute a significant burden of disease [3]. If used effectively, family planning methods may prevent about 32% of maternal and 10% of child deaths in these low resource settings [4]. In addition, strategies to improve health-related outcomes are important, including more years of schooling and spill-over economic outcomes for girls and women [2].

Vasectomy and tubal ligation provide permanent surgical family planning methods for both men and women who do not want more children. However, vasectomy uptake is low, particularly in Africa [3], despite evidence that it is an efficient family planning method with lower costs and fewer complications compared with tubal ligation [5]. Women in African societies are the primary holders of family planning responsibility and use but seldom have primary decision-making power. This is largely attributed to gender roles and power dynamics evident in these societies [6–8]. Deep-rooted cultural practices of women taking responsibility for contraception, often through traditional methods, is coupled with widespread misinformation and assumptions that a vasectomy will damage a man’s pride and masculinity [9]. Even though groups such as health providers and/or development experts may positively promote vasectomy update, previous studies found women were negative about their partners’ having a vasectomy [9–11], often because of a lack of awareness or full information about the procedure [11].

Improving family planning services is a long-standing health policy and strategic priority in Tanzania [12, 13]. This is important to improve the high rates of maternal mortality (556/100,000 live births) and neonatal deaths (25/1,000 live births) by 20%–35% [14]. Family planning in Tanzania remains predominantly a women’s issue, and few men use condoms (15%) [14]. In addition, only about 32% of married Tanzanian women aged 15–49 years use modern family planning methods, such as injectable (13%), implant (7%), and oral (7%) contraception [14]. Of these, about 26% stop complying with contraception within 12 months because of method-related health concerns and side effects [14]. There is a paucity of data regarding vasectomy use in Tanzania, and more broadly across East Africa. In addition, vasectomy is not featured in national health reports, including the latest (2016) demographic health survey [14]. Previous studies reported that less than 1% of the Tanzanian population use vasectomy as a family planning method [15].

There is increasing recognition of the importance of including men in planning and delivering reproductive health services. However, this requires transforming current societal practices and beliefs. As women are the main users of family planning methods, understanding their perceptions and beliefs regarding barriers to vasectomy uptake is needed to support efforts to involve men in reproductive health and promote vasectomy as an effective, low cost option.

## Research team and reflexivity

The research team involved two national and two international colleagues all of whom are PhD qualified nurse-midwives with experience in qualitative research. All data was collected by the principal investigator of this study with the assistance of a female research assistant with previous experience in reproductive health research. The research team had no prior knowledge of the participants of this study. Importantly, however, the team had many insights of pre-understanding of the contextual issues related to family planning in Tanzania. Understanding of local culture and context enhanced the interpretation of data and added to credibility of this study. Of note such pre-understanding might have also lead to researchers presenting their own perceptions rather than solely those of participants, potentially affecting the trustworthiness of the results. This was mitigated by frequent discussion among the local research team and international researchers involved in this study, to ensure a reflected critical position. This helped the research team remain conscious of advantages and potential risks involved in the research process and ensure the results represented barriers as perceived by participating women.

## Methods

### Study design

This study used a qualitative design to capture women’s perceptions and beliefs about vasectomy as a family planning method for their partners/husbands [16], with data gathered via in-depth semi-structured individual interviews. COREQ guidelines informed all steps in the data collection, analysis, and reporting [17].

### Participant selection

The participants included women who were married or cohabiting, had two or more children, and voluntarily agreed to participate in this study. Purposive sampling was used to recruit community-dwelling women who had attended the health facilities and could provide culturally-based rich descriptions of perceptions and beliefs about barriers to vasectomy use. The principal investigator obtained the information regarding these participants at the health facility, and used this to trace women in their communities through community leaders. The principal investigator provided detailed explanation of the study and interacted with the women to agree convenient meeting places and time before the interviews.

### Setting

This study was conducted at health centers and district hospitals in Bagamoyo and Kisarawe, two of six districts in the Pwani region of Tanzania [6]. Tanzania is a low-income East African country with a population of about 44.9 million people, 31.3% of which live in extreme poverty [18]. About 1.1 million people live in Pwani; a majority reside in Bagamoyo (311,740 people) and Kisarawe (101,598 people) districts [18]. Pwani has a rapidly growing population, a high fertility rate, and low family planning use. In Pwani, only 39% of married women use modern family planning methods, with unmet family planning needs estimated at 19% [6].

In Tanzania, healthcare is delivered in public and private facilities at five levels: dispensaries, health centers, and district, regional, and consultant hospitals [13]. Most family planning services are provided at government-owned health facilities across these five levels [13]. District authorities oversee healthcare at a district level [7]. Dispensaries are the community entry point to the health system, with higher levels of care accessed through referral systems [19]. Although health facilities have health committees involving various stakeholders (including community members), actual community involvement in healthcare planning is not yet achieved [7].

### Data collection

A semi-structured interview guide was used to explore participants’ perceptions and beliefs about barriers to vasectomy. The interview guide covered demographic information, knowledge about vasectomy, perceptions and beliefs about the use of vasectomy, and perceived barriers to vasectomy. Twenty interviews were conducted between September 2017 and February 2018 in Kiswahili (the national language) and audio-recorded with participants’ consent. Participants’ non-verbal responses were noted by the interviewer and later connected with findings. The interviews were conducted until data saturation was reached and each lasted 40–60 minutes. Following initial agreement to participate, none of the participants refused to take part or opted to withdraw from the study.

### Data analysis

The audio records were transcribed verbatim by a research assistant. Thematic analysis was used to guide the exploration of manifest and latent expressions in the data [19]. The translation of the interviews from Kiswahili into English was done by a Kiswahili-English linguist to allow non-Kiswahili speaking members of the researcher team to participate in the analysis. This linguist also verified the transcripts and translations against audio-records for truthfulness. All the members of the research team (EP, AM, EH, SB) repeatedly read the transcripts to increase familiarity with the data. Two research team members (EP, AM) using English transcripts performed the initial coding, and constructed sub-themes. The Nvivo version 11 software was used for data analysis, retrieval, easy movement within data, and data storage [16]. A stepwise analysis extracted several codes, themes, and sub-themes (Table 1). Agreement regarding the final themes/sub-themes was reached after discussion among the entire research team.

**Table 1:** Example of qualitative data analysis from interviews with women.

Extract from interview transcripts	Codes	Sub-themes	Theme
…I have never heard or seen someone who practice it (vasectomy). At Chalinze, no one will accept unless they are first given education… *Woman 1*, *Chalinze*	Vasectomy not known	If he was an educated person	Education: whose role and where?
	Acceptance		
…My advice is that, because at least ninety percent have an idea and women gets such education when we visit clinic with our children. Men should be given education about family planning. Not all of them will refuse, there are some of them will agree and practice … *Woman 4*, *Masaki*	Acceptance	Women’s roles and responsibilities in vasectomy uptake	
	Women more knowledgeable		
	Educate men		

## Results and discussion

In total, 20 women from four health facilities (two hospitals and two health centers) in Bagamoyo and Kisarawe districts participated in this study. These participants were from two religious groups (Muslim and Christian). Most women had completed primary-level education only and were engaged in small-scale farming. The number of children per woman ranged from two to six. Table 2 present participant demographic data.

**Table 2:** Participant sociodemographic characteristics.

STUDY SETTING		AGE		RELIGION		LEVEL OF EDUCATION		
District	Health institution	years	Participant number	Muslim	Christian	Primary	Secondary	None/dropped out
Bagamoyo	Chalinze Health Center	20−30	1	4	1	4		1
		31−40	2					
		41−50	2					
	Bagamoyo District Hospital	20−30	1	5	0	4		1
		31−40	3					
		41−50	1					
Kisarawe	Masaki Health Center	20−30	1	3	1	2	0	2
		31−40	3					
		41−50	0					
	Kisarawe District Hospital	20−30	2	4	2	3	3	
		31−40	2					
		41−50	2					
	**Total**		**20**	**16**	**4**	**13**	**3**	**4**

Overall, three main themes about barriers of vasectomy use were identified from data (Table 3), and are discussed below with associated sub-themes. Key points relating to these themes are illustrated by quotes from participants.

**Table 3:** Themes and sub-themes.

Themes	Sub-themes
1. Education: whose role and where?	○ Men’s education level ○ Inadequate knowledge and misinformation
2. Religious beliefs, social pressure and stigma	○ Religious beliefs and vasectomy practice ○ Vasectomy is not good for men with multiple wives
3 Promoting men involvement in family planning	○ Educating men about family planning ○ Women’s role in promoting vasectomy

## Education: Whose role and where?

*Men’s education level* was an important sub-theme that emerged from participants’ comments. Women in this study perceived that a low level of formal education among men in their communities was a major barrier to vasectomy uptake. They believed that men with a higher level of education and from well-educated communities were more likely to accept vasectomy than men with low/no education. They stated that educated men desire to plan families and save money to have a better family life, rather than support big families.

*Some people will see [a man] as breaching customs (if he uses vasectomy), but those who are ahead of us those who are well educated see him as a clever person. Educated people give birth to two, three, or four children, but for us who did not go to school, we go for eight children or ten*. *You’re competing: my neighbor has six kids so I also need six kids*.

Woman 1, Chalinze

Another key sub-theme concerned *inadequate knowledge about vasectomy and misinformation* among women, men and communities. Despite their familiarity with other family planning methods, women perceived that they lacked appropriate knowledge and information about vasectomy. This gap was reflected during the discussion, for instance, some women described vasectomy as unsafe procedure since it involved cutting of men’s reproductive organs which frightened them, their husbands/partners, and the wider community. In contrast, other women believed vasectomy was safe because it was provided at health facilities. As they reflected upon a man who will opt to use vasectomy, one woman stated that:

*He will be despicable eeh! People will see him doing something [vasectomy] which is non-existing*…*They [the community] are not well educated about practicing vasectomy*.

Woman 1, Masaki

Many women in this study were aware that vasectomy did not offer protection against HIV and other infections associated with unprotected sex. Some, women, however had limited understanding and believed that a vasectomy would protect a man from HIV as well as preventing him fathering a child. The number of women that asked if a vasectomy would provide protection against HIV suggested a need for knowledge that vasectomies do not prevent sexually transmitted infections when promoting the procedure. Indicating knowledge gap, a participant commented that:

Vasectomy should prevent both pregnancy and HIV/AIDS

*Woman 3*, *Kisarawe*

Inadequate knowledge and misinformation have also been reported in other parts of Tanzania as barriers to vasectomy uptake [15, 20]. Lack or inconsistent provision of vasectomy, along with lack of infrastructure, limited supplies, lack of trained personnel in health facilities, and non-discussion of vasectomy by healthcare providers is reported to contribute to a low vasectomy uptake [15]. This limits knowledge spread among providers and community. For example, while guidelines and communication materials for female-centered family planning methods are usually available in Tanzania’s rural health facilities, vasectomy-related information is almost nonexistent [15]. While it is important that women have a control of their own bodies and contraception choices, they also desire options for shared responsibility with their partner. The lack of knowledge about vasectomy limits this choice for both men and women.

## Religious beliefs, social pressure and stigma

A key barrier identified by participants concerned the links between *religious beliefs and vasectomy practice*. Some women said their religious beliefs prevented them from engaging in family planning. They perceived men who had a vasectomy as living against “God’s plan.” Some women further explained how their religious beliefs acted as a barrier to family planning, with family planning being seen as “killing living things.” These religious beliefs were considered particularly relevant in rural settings. For example, one woman said:

*Why are you correcting God who planned for the birth? You are contrary to His wish, you are against good values of the religion…In town, you will be seen as a cunning person, but for people like us from rural areas it is an issue*.

Woman 1, Kisarawe

Vasectomy was also perceived as contradicting Islamic beliefs that allow a man to have multiple wives. Participants said that as men could marry more than one wife, each wife may have different preferences regarding the desired number and spacing of children. This meant vasectomy was an unsuitable family planning method. Other participants challenged this view, explaining that men with multiple wives would need new/reversed vasectomy after each wife had the desired number of children. This would allow him to take appropriate care of his children. These women commented that a large number of children contributed to poor living standards and increased the risk that families would be unable to pay school fees.

*There might be a problem between a man and his wives because one wife or two wives may have children, whereas others don’t have*. *It might happen that one wife want a child and not becoming pregnant [after vasectomy]*. *This may cause the marriage to break up because of conflict and misunderstandings in the family*.

*Woman 2*, *Bagamoyo*

Similar to the present study, religious beliefs has been consistently reported in several previous studies conducted in different parts of Tanzania [15, 20, 21] and other countries [10, 22, 23]. Religious beliefs have a powerful influence on individuals’ health behavior and play important roles in local communities including influencing the acceptability of family planning [10]. Therefore, vasectomy uptake remains dependent on powerful religious constructs and how reproductive health matters are defined by communities.

*Social pressure and stigma* was another key sub-theme identified in this study. Participants reported that men with many children were regarded by their community as powerful, proud, and respected. Contrarily, communities named men who had received a vasectomy as “idiots,” “powerless to their wives,” “half men,” and “bushmen.” This highlighted the social power dynamics and gender inequalities that place women in inferior positions.

*The community cannot agree with this issue (vasectomy), they can look at you as a “bush man,” meaning you can be ignored*. *They will say, “for which reasons is this person practicing family planning?” If you practice it [vasectomy], you do it secretly*; *maybe a man could only inform his wife*.

Woman 2, Masaki

*A lot of people judge men for having a vasectomy because it’s such an absurd thing to be done by a man*.

*Woman 2*, *Kisarawe*

The factors associated with men’s acceptance of vasectomy is complex in such contexts. Similar results were reported by other Tanzanian studies that found men believed family planning was solely a woman’s role, thereby justifying their non-involvement [15, 21]. Similarly, a Turkish study that assessed married couples’ opinions about vasectomy reported sociocultural barriers to vasectomy including beliefs that contraception was the woman’s responsibility and sterilized men lost status in society and authority in the family [11]. Although some women in our study highlighted the need for men to practice vasectomy, they were uncertain whether they could ask their partners to undergo the procedure given the associated village-based stigma.

## Promoting men involvement in family planning

*Educating men about family planning* was considered by women as key in increasing uptake in vasectomy use and involving men to reproductive health services. Most women felt that conducting educational sessions and holding meetings with men at their workplaces about family planning (vasectomy included) may promote their uptake of vasectomy. Women also suggested that community health workers could play a role in providing education specifically for men. They explained that men would trust and act on information from medical personnel “heard with their own ears,” and recommended men be given extra explanations and supportive documents (e.g., brochures). Education for men was perceived as complementing health information conveyed by women at home and eventually increased the acceptability and uptake of vasectomies. One woman suggested:

*Encourage men to use contraceptive methods using community health workers*, *or maybe you could announce seminars or meetings about family planning in villages*. *Through these meetings*, *you may encourage men to attend clinics*.

*Woman 4*, *Bagamoyo*

Several studies in low- and middle-income countries [15, 21–23] have reported results consistent with the present study. These studies revealed that orienting men regarding family planning has the potential to increase contraceptive use. The fact that men predominate in decision-making means that up-scaling modern family planning in Tanzania will remain challenging without men’s meaningful engagement in shared decision relating to family size [24].

Another important sub-theme concerned *women’s role in promoting vasectomy uptake*. Because men’s lack of knowledge was seen as a powerful barrier to vasectomy uptake, these women participants perceived themselves as their men’s educators, advisors and supporters in decision-making, and health behaviors regarding vasectomy uptake. Women believed that frequent visits to health facilities with their children might have contributed to them having higher knowledge and awareness of reproductive health matters than men.

*The [woman’s] main role is to educate the father so he can understand it (vasectomy) through action and words too*. *We [women] get this education when visiting clinics with our children*.

Woman 4, Masaki

Women in this study strongly believed that men would accept vasectomy if women shared knowledge, encouraged them to visit health facilities and promised to support them after the procedure. A small family size (which made it possible to pay school fees and save money to meet the family’s long-term goals) was mentioned as a key reason for accepting vasectomy. A participant stated that:

*A woman’s role is to accept and not ignore him, because he will continue with sexual acts as normal*. *You should not ignore your husband, saying he is no longer a man because he practiced vasectomy*. *You need to be humble and respect him so that your marriage will last*.

Woman 1, Bagamoyo

Some women preferred female over male sterilization because of their view that all the responsibility for contraception belonged to women. These women were of the view that this would avoid potential problems faced by men following vasectomy, such as sexuality problems, stigma, and discrimination by the community. Paradoxically, other women emphasized the need to share their family planning responsibilities with men to allow the family to improve health and economic status. Studies conducted in low and middle-income countries reported that women often negatively influenced vasectomy uptake because of the perception that the procedure would lower their men’s reputations [10, 11, 21–24]. In contrast, women in some studies conducted in high-income countries expressed the need for shared responsibility with men, and were increasingly opting for vasectomy as a family planning method [25].

Generally, the results of this study indicate an intersectionality of a complex domains of religion, masculinity, sexuality, power dynamics and gendered roles. Sexuality and ability to produce higher number of children demonstrates masculinity and maintains men’s power in the community. Consequently, vasectomy uptake is negatively affected both at individual and structural levels. Women perceive that education (and health information) may help their male partners to choose financial sustainability over their masculinity practice through vasectomy. Whereas orientation of men to vasectomy as a highly effective and viable male centered contraceptive option is vital, promoting the uptake of this method will require combined efforts and critical analysis of the deep-rooted cultural, religious and socioeconomic barriers through the lenses of intersectionality.

## Conclusions

Participating women perceived vasectomy uptake as being influenced by poor knowledge and misinformation, along with religious and sociocultural barriers. Some women were supportive of men undergoing vasectomy while others were not. Efforts to promote vasectomy and other family planning methods should be directed to mitigating these deep-rooted sociocultural barriers. Orientation of men to vasectomy as a highly effective and viable male centered contraceptive option is vital, and women play an essential role in facilitating the uptake of this method with their partners. Engaging couples in family planning educational sessions may facilitate reproductive health knowledge sharing, including information about vasectomies. This could be enhanced through organized ventures between women and healthcare providers.

### Strengths and limitations

Many women brought their young children to the research interviews, meaning that the flow of the discussion was frequently interrupted by the need to attend childcare concerns. Albeit the sample size, this study provides contextually-based findings, with the potential to be transferable to other rural regional communities within East Africa.

## Supporting information

S1 File Interview guide. (DOCX)
