# Supplementary material for: Religious beliefs, social pressure, and stigma: Rural women’s perceptions and beliefs about vasectomy in Pwani, Tanzania
Source: PLoS One. 2020 Mar 20;15(3):e0230045. doi: 10.1371/journal.pone.0230045 (PMC7083335; doi:10.1371/journal.pone.0230045)
Supplement: S1 File — (DOCX) [file pone.0230045.s001.docx]

**INTERVIEW GUIDE**

**Rural women’s perceptions and beliefs about vasectomy in Pwani, Tanzania**

Participant number:

Tribe:

Age:

Number of children:

Work:

Religion:

Level of education:

Date:

| **Interview Guide with Women** | **Theme** |
| --- | --- |
| Thank you for accepting to participate in this interview. My name is……Today, I would like to discuss about your overall perceptions and beliefs on family planning utilization. This interview may take about one hour. | BEGINNING |
| Have you read the information sheet?  Do you understand that you can change your mind about being involved with the study at any time?  Are you OK to sign this form to say that you’ve understood what is going to happen? | CONSENT |
| Is it ok if I record the interview?  Have you got any questions about the study?  If you want to stop the interview, please let me know and we will stop immediately | BEFORE WE START |
| The reason for talking to you today is to find out about your perceptions and beliefs regarding vasectomy. |  |
| Can you tell me a bit about yourself?   - *Marital status, Family and herself* - *Family planning methods used* | TELL ME A BIT ABOUT YOURSELF |
| Can you share with me about what you know regarding family planning methods used by men?   - *Own perception and belief about vasectomy* - *Perceptions about men views of vasectomy/reproductive services?* | Knowledge, perceptions and beliefs |
| Is there anything else you would like to tell me about vasectomy and generally concerning family planning methods for men? | ENDINGS |
| Other people have said………  Is that your experience?  I’d now like to move on and talk about your experiences ……  You just mentioned …. and that brings me on to another one of my questions  Can you give me an example of………..  Some people I’ve interviewed said ……. why do you think that might be?  I just wanted to clarify …..  You mentioned that …… could you explain why? | ADDITIONAL PROMPTS |
